# Nitrative Stress Participates in Endothelial Progenitor Cell Injury in Hyperhomocysteinemia

**DOI:** 10.1371/journal.pone.0158672

**Published:** 2016-07-08

**Authors:** Yu Dong, Qi Sun, Teng Liu, Huanyuan Wang, Kun Jiao, Jiahui Xu, Xin Liu, Huirong Liu, Wen Wang

**Affiliations:** 1 Department of Physiology and Pathophysiology, School of Basic Medical Sciences, Capital Medical University, Beijing, China; 2 Beijing Key Laboratory of Metabolic Disorders Related Cardiovascular Diseases, Capital Medical University, Beijing, China; Centro Cardiologico Monzino, ITALY

## Abstract

In order to investigate the role of nitrative stress in vascular endothelial injury in hyperhomocysteinemia (HHcy), thirty healthy adult female Wistar rats were randomly divided into three groups: control, hyperhomocysteinemia model, and hyperhomocysteinemia with FeTMPyP (peroxynitrite scavenger) treatment. The endothelium-dependent dilatation of thoracic aorta *in vitro* was determined by response to acetylcholine (ACh). The histological changes in endothelium were assessed by HE staining and scanning electron microscopy (SEM). The expression of 3-nitrotyrosine (NT) in thoracic aorta was demonstrated by immunohistochemistry and immunofluorescence, and the number of circulating endothelial progenitor cells (EPCs) was quantified by flow cytometry. Hyperhomocysteinemia caused significant endothelial injury and dysfunction including vasodilative and histologic changes, associated with higher expression of NT in thoracic aorta. FeTMPyP treatment reversed these injuries significantly. Further, the effect of nitrative stress on cultured EPCs *in vitro* was investigated by administering peroxynitrite donor (3-morpholino-sydnonimine, SIN-1) and peroxynitrite scavenger (FeTMPyP). The roles of nitrative stress on cell viability, necrosis and apoptosis were evaluated with 3-(4,5-dimethylthiazol)-2,5-diphenyl tetrazolium (MTT) assay, lactate dehydrogenase (LDH) release assay and terminal deoxynucleotidyl transferase dUTP nick-end labeling (TUNEL) assay, respectively. Also, the phospho-eNOS expression and tube formation in Matrigel of cultured EPCs was detected. Our data showed that the survival of EPCs was much lower in SIN-1 group than in vehicle group, both the apoptosis and necrosis of EPCs were much more severe, and the p-eNOS expression and tube formation in Matrigel were obviously declined. Subsequent pretreatment with FeTMPyP reversed these changes. Further, pretreatment with FeTMPyP reversed homocysteine-induced EPC injury. In conclusion, this study indicates that nitrative stress plays a role in vascular endothelial injury in hyperhomocysteinemia, as well as induces endothelial progenitor cell injury directly.

## Introduction

Homocysteine (Hcy) is an intermediate product of methionine metabolism *in vivo*. The World Health Organization stated that the fasting plasma homocysteine levels in healthy adults range from 5 to 15 μmol/L. Elevated plasma homocysteine is associated with the development of coronary artery disease, myocardial infarction, stroke and peripheral vascular disease [1–5]. Hyperhomocysteinemia (HHcy) (plasma homocysteine levels more than 15 μmol/L) is an established risk factor for coronary heart disease and other cardiovascular diseases [6,7]. Homocysteine may directly or indirectly cause vascular endothelial damage and promote the proliferation of vascular smooth muscle cells [8]. Endothelial dysfunction was exhibited in multiple hyperhomocysteinemic animal models (including mice [9], rats [10], and primates [11]). However, the underlying mechanism is not fully understood yet. Recent studies have indicated that hyperhomocysteinemia improved the activity of inducible nitric oxide synthase (iNOS) and increased the generation of nitric oxide (NO), and induced the production of ROS, which greatly enhanced the formation of peroxynitrite (ONOO^-^) [12]. Peroxynitrite-mediated nitrative stress leads to severe damage to proteins, lipids, and DNA [13], resulting in cell damage or apoptosis and cytotoxicity. 3-nitrotyrosine (NT) formation has been used extensively as a footprint for the generation of peroxynitrite *in vivo* via nitration of tyrosine residues on proteins. The endothelial progenitor cells (EPCs) play a crucial role in repairing the injured endothelium and re-endothelialization [14]. The amount and bioactivity of endothelial progenitor cells have became an important indicator of vascular endothelial function and risk assessment in cardiovascular disease [15,16]. What’s the effect of elevated nitrative stress on endothelial progenitor cells in hyperhomocysteinemia? In the current study, by using the hyperhomocysteinemic rats model *in vivo* and primarily cultured endothelial progenitor cells *in vitro*, we would investigate the role of nitrative stress in hyperhomocysteinemia-induced endothelial dysfunction, mainly focused on the indicator of endothelial repair-endothelial progenitor cells.

## Materials and Methods

### Animals

The experimental procedures were compliant with the Guiding Principles in the Use and Care of Animals published by the National Institutes of Health (USA) and were approved by the Institutional Animal Care and Use Committee of Capital Medical University (China). Animals were provided by Vital River, License: SCXK (Beijing), 2012–0001. Before the start of the experiments, all animals were housed in a room under a 12 h light/dark cycle and controlled humidity and temperature, with free access to food and water. The Wistar rats were performed under pentobarbital sodium (150 mg/kg) anesthesia to reduce suffering. Once the experiment was completed, all the Wistar rats were euthanized by decapitated at the guillotine (a physical method was suggested by AVMA Guidelines on Euthanasia). 6-week-old female C57BL/6 mice for EPCs culture were sacrificed by cervical dislocation.

The 30 healthy, adult female Wistar rats (SPF grade) were randomly divided into 3 groups: (1) control, n = 10, females; (2) hyperhomocysteinemia model, n = 10, females; (3) hyperhomocysteinemia treated with FeTMPyP (peroxynitrite scavenger, Cayman), n = 10, females. All control rats received normal diet and the other two groups received rat chow containing 2.5% methionine. HHcy + FeTMPyP group received FeTMPyP (3 mg/kg) weekly via intraperitoneal injection at 10–16 weeks. The rats were sacrificed after 16 weeks.

### Plasma homocysteine assays

Carotid artery was cannulated and procoagulant tubes were used to obtain serum. The tube was centrifuged at 3000 rpm, 4°C, for 10 min. The supernatant was used for homocysteine detection by enzyme-linked immunosorbent assay (ELISA).

### Endothelium-dependent vasodilatation

After rats were euthanized with pentobarbital sodium (150 mg/kg), fresh thoracic aortas were isolated from the chest and transferred into ice-cold and oxygenated HEPES buffer (mM: NaCl, 144; KCl, 5.8; MgCl_2_·6H_2_O, 1.2; CaCl_2_, 2.5; Glucose, 11.1; HEPES, 5; pH 7.38–7.40). The thoracic aortas were isolated from adipose and connective tissues and cut into arterial rings of 3 to 4 mm in length. These segments were attached to two wires (40 mm) connected to an isometric force transducer (DMT610M, Danish Myo Technology), containing oxygenated (95% O_2_, 5% CO_2_) HEPES buffer at 37°C. The artery segments were stretched eventually to an optimal resting tension of 2.0g, which was maintained throughout the experiment. The segments were equilibrated for 2 h before vasorelaxation measurements were performed.

After the equilibration period, the artery segments were exposed to HEPES buffer containing 60 mM potassium (mM: NaCl 29.8, KCl 120.1, MgCl_2_·6H_2_O 1.2, CaCl_2_ 2.5, Glucose 11.1, HEPES 5, pH 7.35–7.45) until reproducible contractile responses were obtained. After washing with HEPES buffer, segments of thoracic aortas were pre-contracted with norepinephrine (NE, 10^−6^ mol/L). Once a stable contraction was achieved, increasing concentrations of acetylcholine (ACh, 10^−9^–10^−5^ mol/L) were added to the chamber to obtain cumulative concentration-response curves. Subsequently, the thoracic aortas were washed, balanced to basic tension and the above steps were repeated. Setting NE (10^-6^mol/L) the maximal contraction amplitude as 100%, changes in vascular tension were reflected by the percentage of ACh-induced vasodilation and NE-induced maximum contraction.

### Scanning electron microscope observation

Thoracic aortas were sliced into 3-mm-long sections approximately. The wall was slit carefully with ophthalmic scissors, unfolded on a filter paper, and transferred to 3% glutaraldehyde for 2 h. Segments of thoracic aortas were washed with 0.1 M PB buffer for 10 min 3 times, and stored in 0.1M PB solution at 4°C. Before observation, samples were generally fixed with osmium, dehydrated, dried and sprayed with gold. The scanning electron microscope was used, setting the voltage to 25kV.

### Hematoxylin-Eosin staining

Thoracic aortas were fixed in 4% paraformaldehyde, and embedded in paraffin using tissue embedding machine (Leica EG 1150 H, Germany). The arteries were sectioned in the vertical plane into 4-μm-thick fragments. Briefly, sections were prepared orderly by dewaxing, staining and dehydration. The morphology of endothelial cells was observed under the microscope (Leica, Germany).

### Measurement of 3-nitrotyrosine (NT)

Immunohistochemical staining was used to examine 3-nitrotyrosine expression. Briefly, thoracic aortas were removed and stored in 4% paraformaldehyde for less than 48 h. Fixed thoracic aortas were dehydrated and embedded in paraffin, and 4-μm-thick sections were obtained and mounted on glass slides. Antigen was retrieved using a microwave method (citric acid buffer, PH 6.0). Endogenous catalase was inactivated with 3% hydrogen peroxide for 10 min at room temperature. The sections were stained with primary antibody (anti-3-nitrotyrosine (NT), Abcam) at 4°C overnight and peroxidase-conjugated affinipure secondary antibody (Santa Cruz) at 37°C for 30 min, consecutively. 3-Nitrotyrosine was detected with diaminobenzidine (DAB). Protein quantification was performed using ordinary optical microscope (Leica, Germany).

Immunofluorescence was also performed as described previously followed by visualization under an inverted fluorescence microscope (OLYMPUS, Japan).

### Flow cytometry

In brief, 100 μL of peripheral blood was incubated with a three-fold volume of red cell lysate on ice for 15 min. After centrifugation of 450 g at 4°C for 10 min, the supernatant was discarded and two-fold volume of red cell lysate was centrifuged again to precipitate the white blood cells. Samples were then incubated with 400 μL PBS buffer containing 2 μL fluorescein isothiocyanate (FITC)-conjugated CD34 (1:200, Santa Cruz) or phycoerythrin (PE)-conjugated CD31 (1:200, Santa Cruz) antibodies in the dark and were subjected to flow cytometry using the LSRFortessa Flow cytometer (Becton Dickinson, New Jersey, USA). Cells positive for CD34^+^/CD31^+^ within the lymphocyte population were characterized as EPCs.

### Culture and treatment of EPCs

Density gradient centrifugation was used to isolate mouse bone marrow progenitor cells. EPCs surface markers VE-Cadherin (Santa Cruz), CD133 (Santa Cruz), VEGFR-2 (Santa Cruz) (three primary antibody dilutions of 1:100, and a fluorescent secondary antibody dilution of 1:500) and uptake of Dil-ac-LDL (Molecular Probes) as well as binding function with FITC-UEA-1(Sigma-Aldrich) were identified.

Second to fourth generation cells were selected in the experiment. Cells in 96-test plate were randomly divided into: control group, SIN-1 hydrochloride (peroxynitrite donor, 3-Morpholinosydnonimine hydrochloride, Sigma-Aldrich, USA) treatment and SIN-1+FeTMPyP (10 μmol/L) groups. Treatment group was supplemented with different concentrations of SIN-1 (0, 200, 400, 600, 800, and 1000 μmol/L) with 1% FBS for 24 h. The appropriate concentration for subsequent experiments was determined by MTT [3-(4, 5-dimethylthiazol-2-yl)-2,5-diphenyltetrazolium bromide] assay, based on the cleavage of tetrazolium salts by mitochondrial succinate reductase in viable cells to form formazan dye. After treatments, MTT (5 mg/mL) was added to each well and incubated for 4 h at 37°C and the formazan crystals were dissolved in DMSO. The absorbance was measured at 490 nm and the cell viability expressed as optical density with reference to the control. Subsequently, LDH and TUNEL methods were performed to detect the necrosis and apoptosis. Cells were incubated with homocysteine (2000 μmol/L, 24 h) or pretreated with FeTMPyP (10 μmol/L), to evaluate the cell viability by MTT.

### Western blot

Phospho-eNOS expression in EPCs was measured by western blot. Total proteins were obtained from cells using cell lysis buffer and were quantified with BCA Protein Assay Kit (Thermo Scientific). 50 μg of proteins were subjected to 8% SDS-PAGE and then transferred onto a PVDF membrane. Primary antibodies against anti-phospho-eNOS (Ser 1177) monoclonal (1: 1000, EnoGene) and anti-β-actin monoclonal (1: 1000, Cell Signaling Technology) were incubated at 4°C overnight. Next, the membranes were blotted with the horseradish peroxidase-conjugated anti-rabbit IgG for 1 h (1: 3000, Beijing Zhongshan Golden Bridge Biotechnology). The membranes were washed and proteins were detected using a Western Blotting Luminol Reagent systerm and autoradiography.

### Matrigel Assay

Thaw the Matrigel (BD) at 4°C overnight preventing it to room tempreture. Add 70 μL of Matrigel to each well of 96-well plate and then leave the Matrigel to solidify at 37°C for 30 minutes. 3×10^4^ EPCs were seeded on top of the Matrigel layer in 200 μL of culture medium. Next, the cells were incubated at 37°C in 5% CO_2_ [17,18]. Subsequently, the tube network was observed by brightfield microscope during 12 h period. These images were analyzed using MetaMorph Software.

### Statistical analysis

SPSS v13.0 was used for all statistical analysis and all data were presented as the means ± standard deviation (SD). Repeated measures ANOVA followed by Bonferroni post hoc test was used to assess the statistical differences of vascular responses to ACh among groups. One-way analysis of variance (ANOVA) followed by Bonferroni post hoc test was used to analyze the others. *P* value < 0.05 was considered significant.

## Results

### 1. The plasma homocysteine level was significantly increased

**Fig 1:**
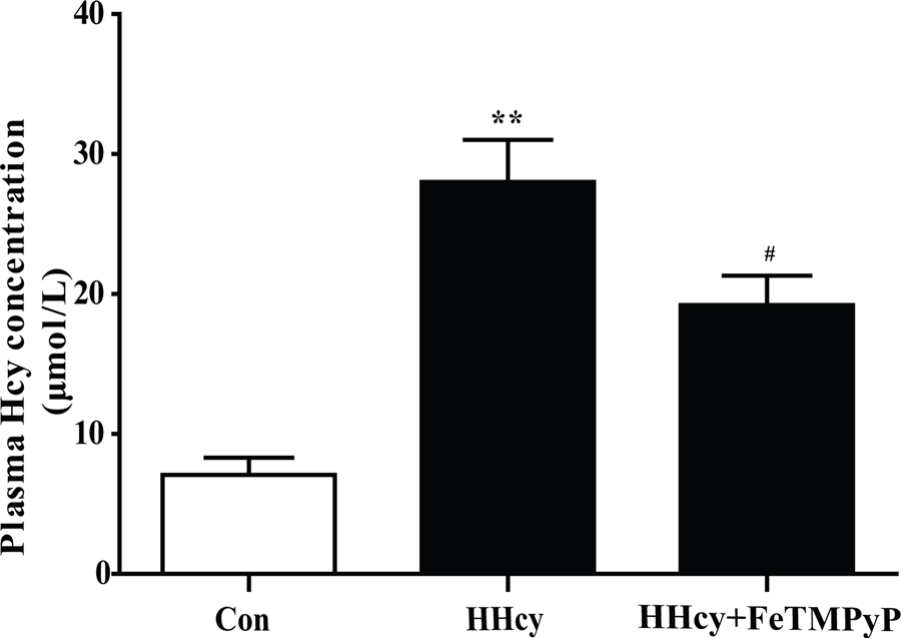
Assay of plasma homocysteine level. The plasma homocysteine level of rats was significantly increased after they were fed with 2.5% methionine diet for 16 weeks. Treatment with ONOO^-^ scavenger FeTMPyP decreased the homocysteine level. Data were expressed as mean±SD; n = 6–10. **P<0.01 versus Control; ^#^*P<*0.05 versus HHcy. Control: normal rats; HHcy: hyperhomocysteinemia; FeTMPyP: ONOO^-^ scavenger.

The Wistar rats were fed with 2.5% methionine diet for 16 weeks to establish rat hyperhomocysteinemia model. The bar chart revealed that the level of plasma homocysteine was significantly increased, indicating that the rat hyperhomocysteinemia model was reliable. Anti-peroxynitrite treatment significantly decreased plasma homocysteine level (Fig 1).

### 2. Both endothelium-dependent vasorelaxation and morphology of thoracic aortas changed in hyperhomocysteinemia rats, and anti-peroxynitrite treatment significantly alleviated endothelial injury

**Fig 2:**
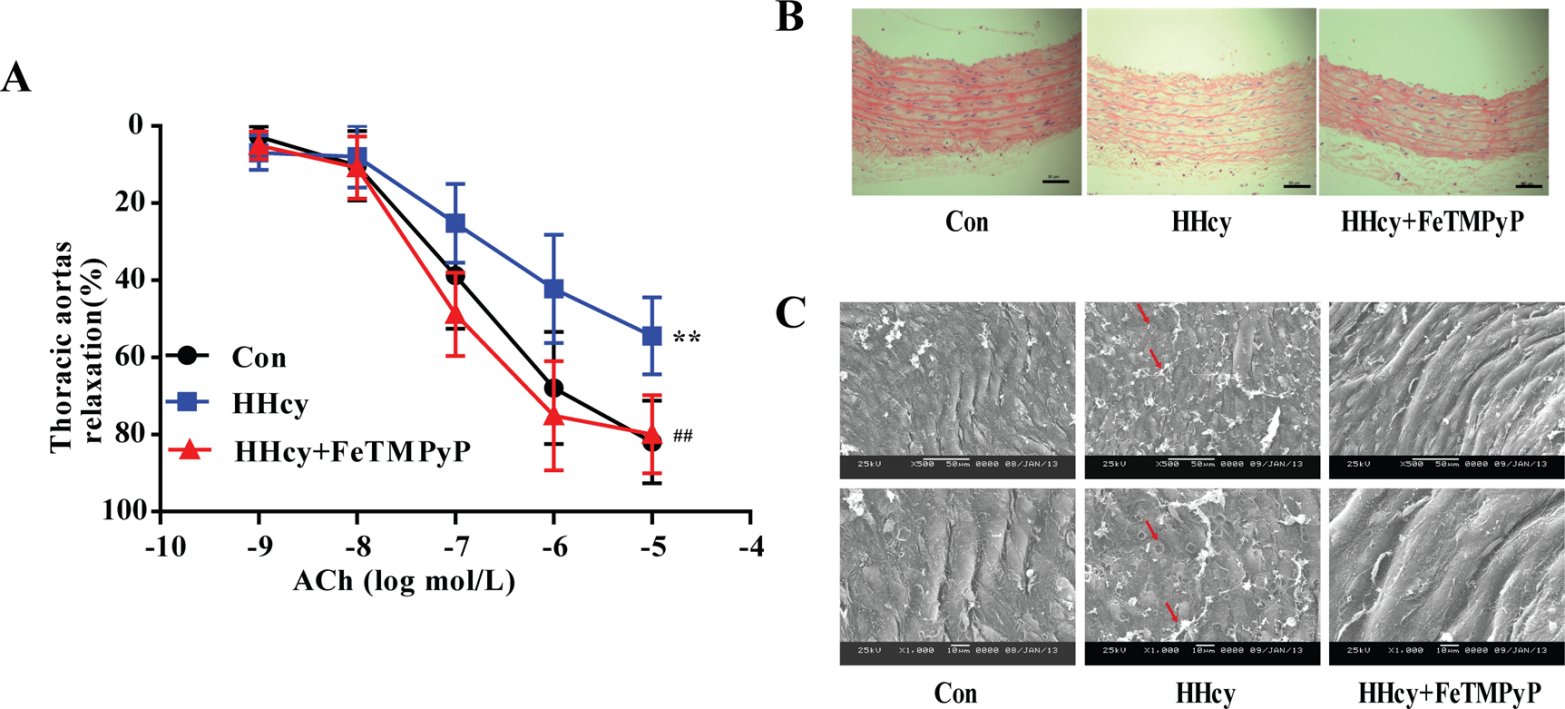
Endothelium-dependent vasorelaxation and morphology change of thoracic aorta. Both endothelium-dependent vasorelaxation and morphology of thoracic aorta were measured in hyperhomocysteinemia rats, and anti-peroxynitrite treatment significantly alleviated endothelial injury induced by hyperhomocysteinemia. (A) ACh-induced endothelium-dependent vasorelaxation. Data were expressed as mean±SD; n = 6–10. Differences between the groups were assessed by repeated measures ANOVA followed by Bonferroni post hoc test. **P<0.01 versus Control; ^##^*P<*0.01 versus HHcy. (B) HE staining. Bar represented 50 μm; (C) Scanning electron microscopic observation of thoracic aortas in which the arrows showed the damaged endothelial cells and fibrin-like material. Bar represented 50/10 μm.

In order to probe the change in vascular function with hyperhomocysteinemia, we detected the ACh-induced vasorelaxation in the thoracic aortas. ACh is an endothelium-dependent vasodilator that relaxes NE-induced vasoconstriction in control rats. Endothelium-dependent vasorelaxation in hyperhomocysteinemia rats was lower than in control rats (*P*<0.01). FeTMPyP, a peroxynitrite scavenger, increased endothelium-dependent vasorelaxation compared with hyperhomocysteinemia rats (Fig 2A). The endothelium of thoracic aortas in hyperhomocysteinemia rats was apparently damaged, although the cell arrangement of smooth muscle cells was not altered. The injury to thoracic aortas in control and HHcy + FeTMPyP group was not obvious. The endothelium was basically complete, and the smooth muscle cells were normally arranged (Fig 2B). Additionally, endothelial cells of hyperhomocysteinemia rats were atrophied, deformed, and even desquamated. The damaged endothelial cells and the adhered fibrin-like material were visible (Fig 2C).

### 3. 3-nitrotyrosine expression in thoracic aortas was elevated in hyperhomocysteinemia rats, and anti-peroxynitrite treatment lowered the NT expression

**Fig 3:**
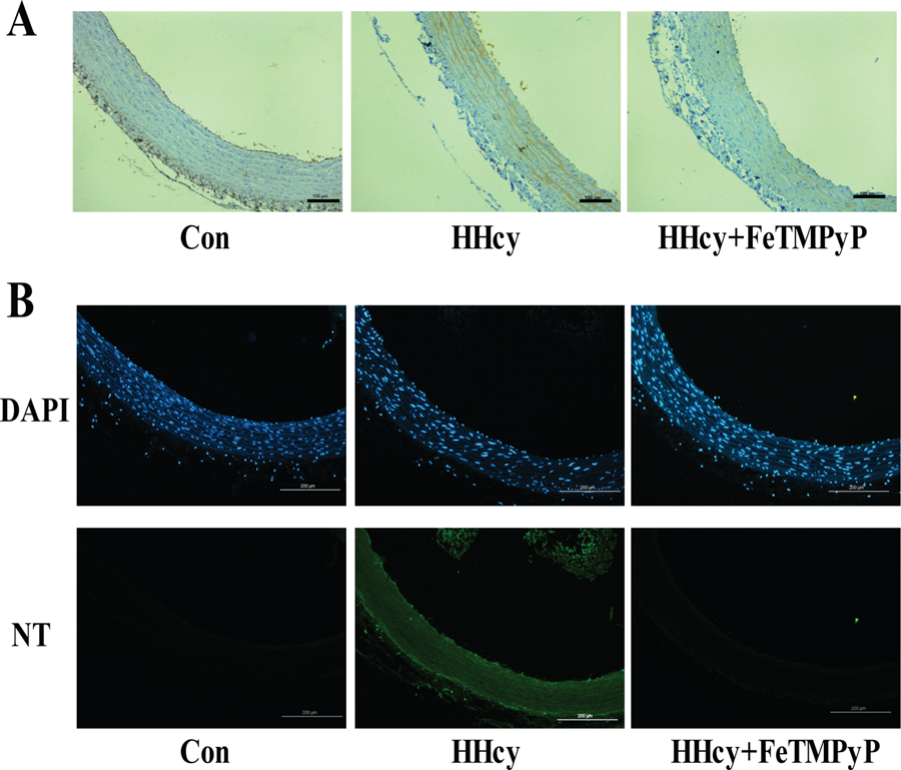
NT expression in thoracic aortas. The level of NT in thoracic aortas was lifted in response to homocysteine which was inhibited by FeTMPyP. (A) Immunohistochemistry staining. Bar represented 50 μm; (B) Immunofluorescence staining. Bar represented 100 μm. n = 3. HHcy: hyperhomocysteinemia; FeTMPyP: ONOO^-^ scavenger; NT: 3-nitrotyrosine.

Due to the instability of preoxynitrite and difficulty of direct detection in vivi, 3-nitrotyrosine (NT) is known as a footprint of preoxynitrite production. The expression of NT in thoracic aortas in hyperhomocysteinemia group was significantly higher than in control rats. FeTMPyP significantly decreased NT expression compared to hyperhomocysteinemia rats (Fig 3).

### 4. EPCs amount in peripheral blood was reduced in hyperhomocysteinemia rats, and anti-peroxynitrite treatment reversed this reaction

**Fig 4:**
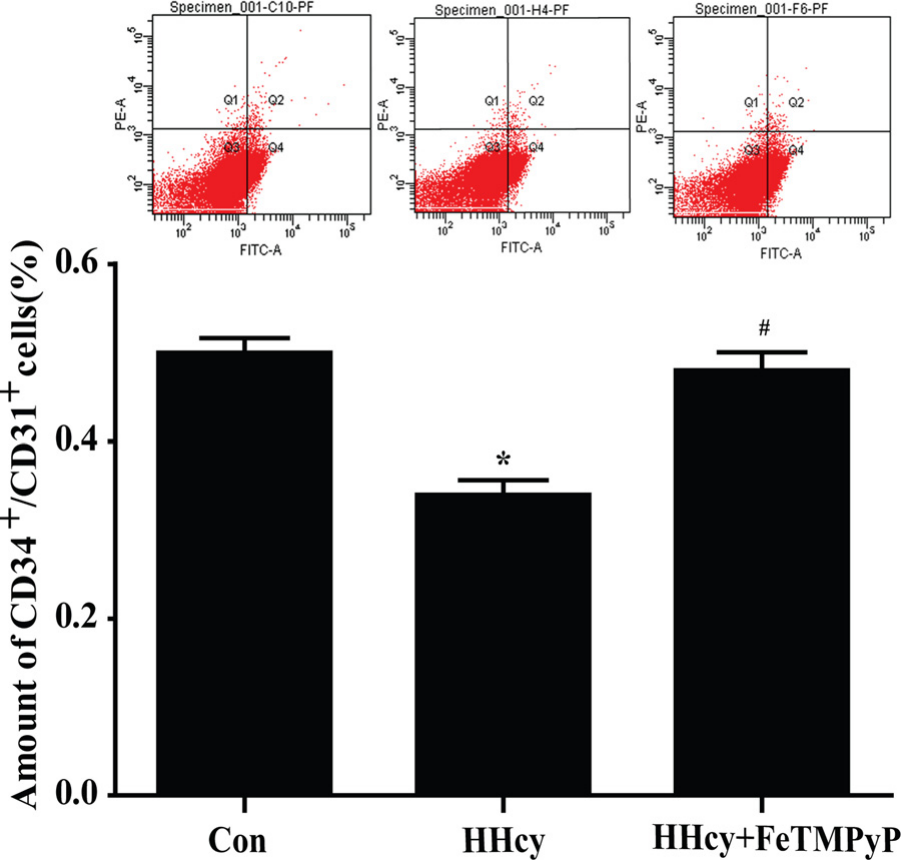
Flow cytometry analysis of endothelial progenitor cells (EPCs). The amount of circulating EPCs (CD31^+^/ CD34^+^) in peripheral blood was significantly reduced in hyperhomocysteinemia rats, and anti-peroxynitrite treatment reversed this reaction. Data were expressed as means±SD; n = 4–6. *P<0.05 versus Control; ^#^*P<*0.05 versus HHcy. HHcy: hyperhomocysteinemia; FeTMPyP: ONOO^-^ scavenger; EPCs: endothelial progenitor cells.

The number of circulating EPCs was assessed by flow cytometry. The scatter plots suggested a significant reduction of circulating EPCs (CD34^+^/CD31^+^) amount in peripheral blood of hyperhomocysteinemia rats compared with control. FeTMPyP significantly increased the amount of circulating EPCs (Fig 4).

### 5. Identification of bone marrow-derived EPCs

**Fig 5:**
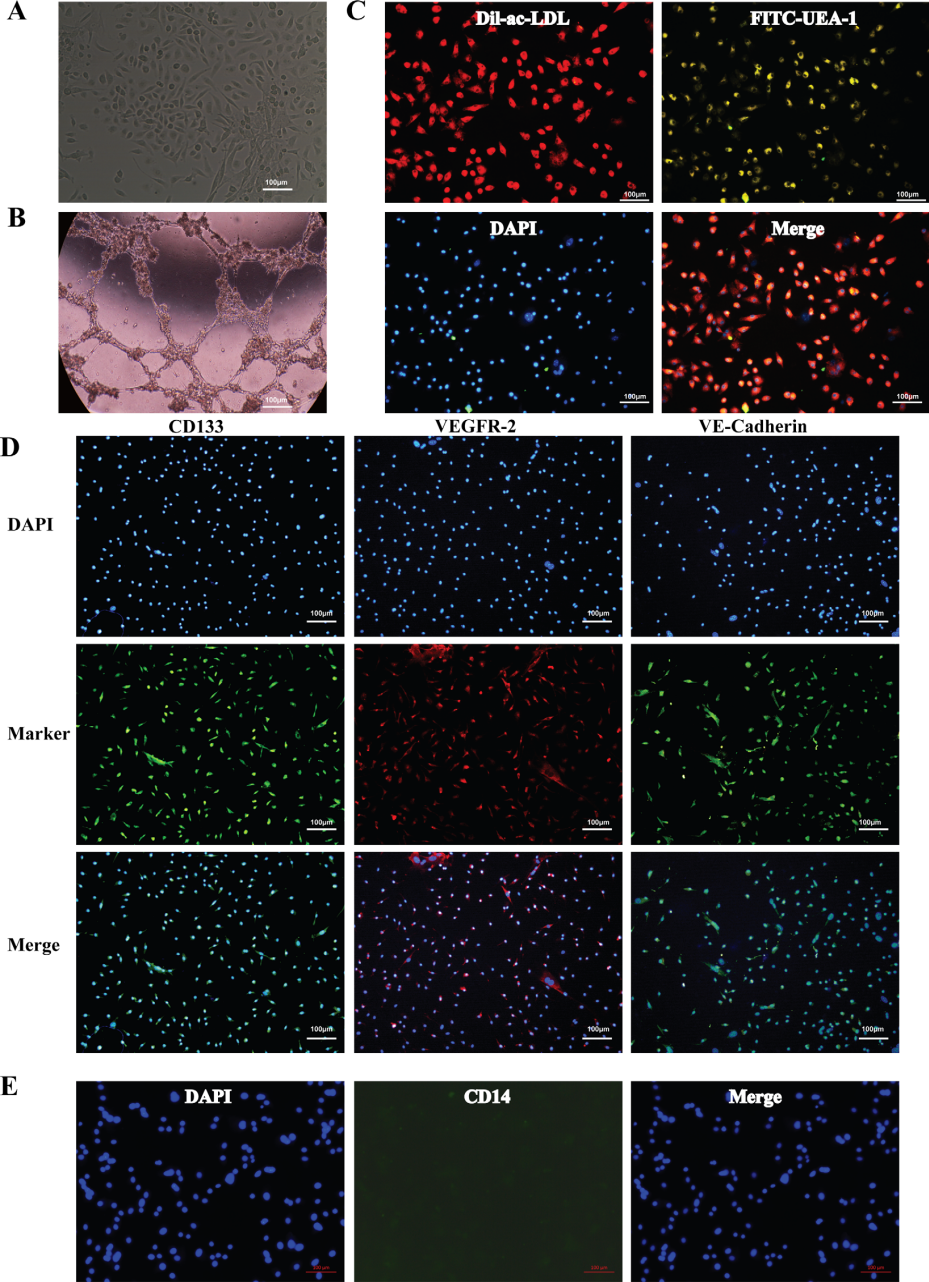
Identification of mouse bone marrow-derived endothelial progenitor cells (EPCs). (A) Microscopic photographs of EPCs. (B) EPCs Matrigel assay. (C) DiI-ac-LDL uptaking and UEA-1 binding of EPCs were positive with immunofluorescence staining. (D) The Markers of EPCs [CD133, VEGFR-2 and VE-Cadherin] were positive with immunofluorescence staining. (E) The marker of monocytes. Bar represented 100 μm.

To explore the effect of nitrative stress on EPCs in vitro, we cultured endothelial progenitor cells from mouse bone marrow. EPCs were identified as well-circumscribed monolayers of cells with cobblestone morphology under inverted microscopy (Fig 5A). The uptake of Dil-ac-LDL by endothelial progenitor cells, FITC-UEA-1 binding (Fig 5C), surface marker (VE-Cadherin, VEGFR-2), stem cell marker CD133 (Fig 5D) were positive and the potential of tube formation (Fig 5B) was tested, which manifesting a successful culture of endothelial progenitor cells. Furthermore, in order to rule out the possibility of monocytes contamination in vitro cultured EPCs, we detected the surface marker (CD14) of monocytes. There was no CD14^+^ positive cell appeared in cultured EPCs.

### 6. Peroxynitrite donor SIN-1 induced EPCs injury *in vitro*, and anti-peroxynitrite pretreatment prevented the adverse effect of SIN-1

**Fig 6:**
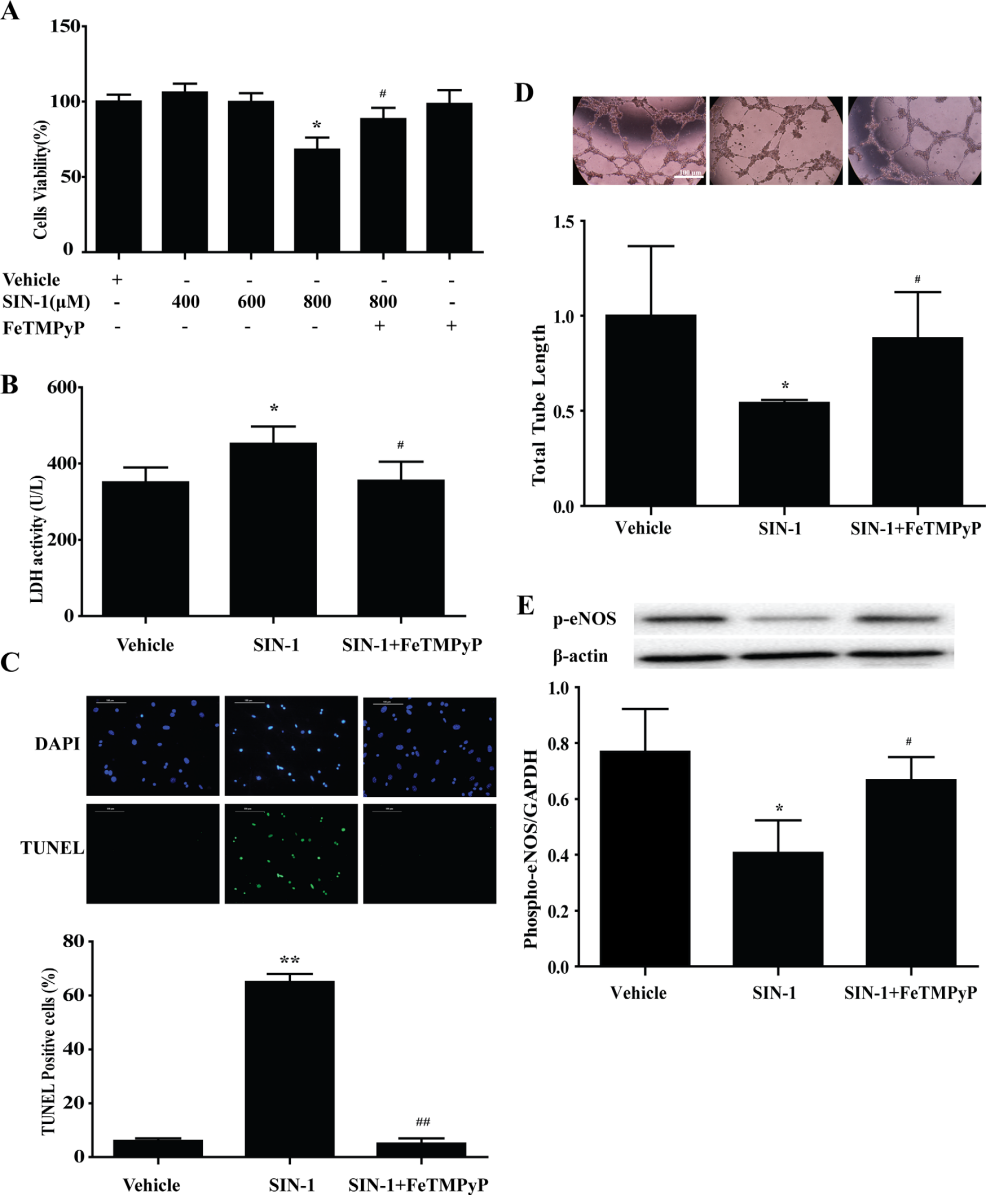
Peroxynitrite donor SIN-1 induced EPCs injury *in vitro*, and anti-peroxynitrite pretreatment prevented the adverse effect of SIN-1. (A) SIN-1 (800 μmol/L) significantly reduced the cell survival rate of EPCs, and was selected as the effective stimulation concentration for further study. Pretreatment with peroxynitrite scavenger FeTMPyP (10 μmol/L) prevented the adverse effect of SIN-1 on the survival of EPCs. The LDH activity (B), TUNEL staining (C) were increased as well as the total tube length (D), the expression of phospho-eNOS (E) were decreased with SIN-1 treatment, which could be reversed by FeTMPyP. *P<0.05, **P<0.01 versus Vehicle; ^#^*P<*0.05, ^##^*P<*0.01 versus SIN-1 (800μmol/L). n = 5. SIN-1: ONOO^-^ donor; FeTMPyP: ONOO^-^ scavenger.

MTT assay was used to determine the cytotoxicity of different concentrations of peroxynitrite donor SIN-1 on the EPCs. SIN-1 (800 μmol/L) significantly reduced the cell survival rate of EPCs, and was selected as the effective stimulation concentration for further study. Pretreatment with peroxynitrite scavenger FeTMPyP (10 μmol/L) prevented the adverse effect of SIN-1 on the survival of EPCs (Fig 6A). LDH activity demonstrated apparently increased necrosis in EPCs following SIN-1 treatment, which was inhibited by FeTMPyP pretreatment (Fig 6B). TUNEL staining showed a similar trend of apoptosis (Fig 6C). And, treatment with FeTMPyP reversed the phospho-eNOS expression and the ability of tube formation induced by SIN-1(Fig 6D and 6E).

### 7. Expression of NT in EPCs was elevated following SIN-1 treatment which was revered by FeTMPyP

**Fig 7:**
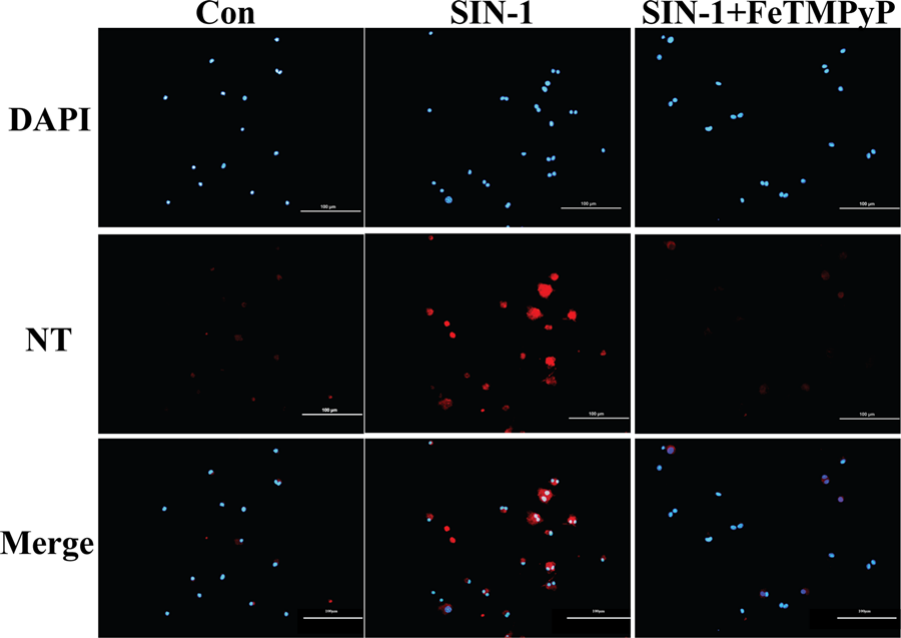
NT expression in EPCs. Cells were treated with SIN-1 (800 μmol/L) or FeTMPyP (10 μmol/L) for 24 h. The level of NT was determined using immunofluorescence. The results prompted that EPCs NT expression was increased in the presence of SIN-1 which could be reduced by FeTMPyP. Bar represented 100 μm. SIN-1: ONOO^-^ donor; FeTMPyP: ONOO^-^ scavenger.

Immunofluorescence was employed in the examination of NT expression in EPCs. The results revealed a significantly elevated expression of NT in endothelial progenitor cells following SIN-1 treatment. In contrast, hardly any fluorescence signal of NT was observed with FeTMPyP treatment compared with SIN-1 treatment alone (Fig 7).

### 8. Anti-peroxynitrite pretreatment reversed the homocystein- induced EPCs injury

**Fig 8:**
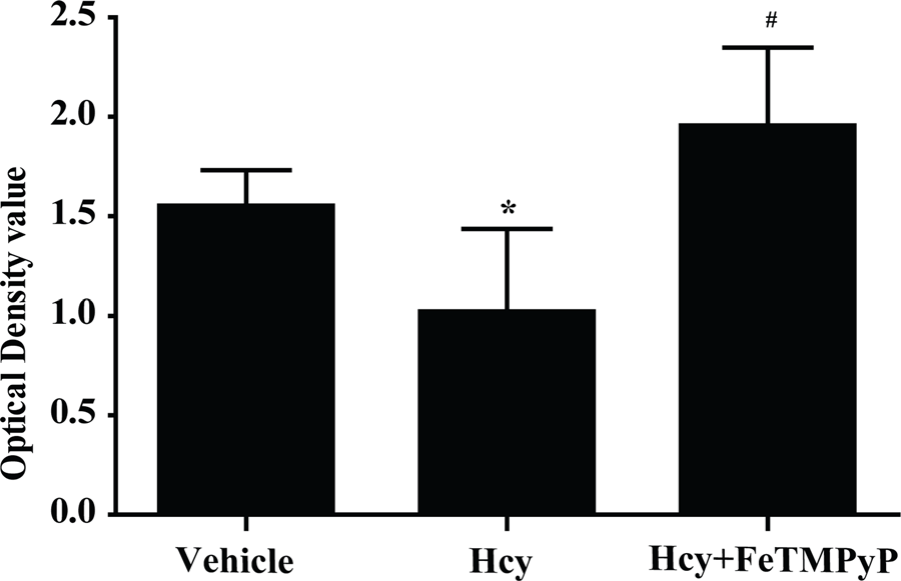
MTT assay of EPCs on the role of homocysteine. The survival rate of homocysteine group was significantly decreased, and improved significantly after pretreatment with FeTMPyP. *P<0.05 versus Vehicle; ^#^*P<*0.01 versus Hcy. n = 5–8. SIN-1: ONOO^-^ donor; FeTMPyP: ONOO^-^ scavenger.

The preceding experiments showed that peroxynitrite induced injury of EPCs *in vitro*. We next determined the effect of homocysteine on EPCs. MTT was applied after treating with homocysteine for 2 h in 2000 μmol/L. There was a significant decrease of EPCs survival rate in homocysteine group, and pretreatment with FeTMPyP significantly improved the survival rate of EPCs (Fig 8).

## Discussion

Different degrees of endothelial dysfunction have been reported in a range of experimental hyperhomocysteinemia animal models. However, the underlying mechanism of endothelial dysfunction associated with elevated plasma homocysteine remains poorly understood [19,20]. In this study, we introduced the effect of nitrative stress on vascular endothelial injury during the development of hyperhomocysteinemia.

Nitric oxide (NO) represents important signal molecules in various physiological conditions, such as vasodilatation, cell growth and angiogenesis [21,22,23]. However, endothelium-dependent vasodilation was reduced by decreased NO bioavailability, which was related to elevated peroxynitrite in pathological conditions [24,25]. The 3-nitrotyrosine (NT) is a marker of nitrative stress [26,27], which is generated by the reaction between tyrosine residues or free tyrosine and peroxynitrite. Recent studies have reported that Hcy increased the synthesis of inducible nitric oxide synthase (iNOS) and ROS [28], generating large quantities of peroxynitrite, which were consistent with our present results that the NT expression of thoracic aorta in hyperhomocysteinemia rats was significantly increased. Peroxynitrite decomposition products contributed to pathological outcomes involving tissue damage [29], tyrosine nitration of protein [30], as well as superoxide dismutase (SOD) inactivation [31,32]. These reports were in keep with our observations that anti-peroxynitrite treatment mitigated endothelial dysfunction significantly. Endothelium-dependent vasodilation dysfunction results from the excessive degradation of NO in hyperhomocysteinemia following inactivation of SOD by high levels of peroxynitrite [33]. Removal of peroxynitrite partially or completely improves vasodilation as SOD converts superoxide into NO [34]. Further, in hyperhomocysteinemia, oxidation of cysteine by endothelial nitric oxide synthase (eNOS) results in decreased NO activity [35]. There are reports that nitrative stress induces alterations and inactivation in potassium conductivity, leading to vasodilation dysfunction mediated by endothelium-derived hyperpolarizing factor [36]. Thus, our foregoing studies have shown a potential relationship between hyperhomocysteinemia-mediated endothelial dysfunction and nitrative stress.

Endothelial progenitor cells (EPCs) from peripheral blood monocytes have been propagated as a novel approach in the diagnosis and follow-up of vascular developments in cardiovascular diseases and endothelial dysfunction [37–40]. Decline in circulating EPCs may be linked to cardiovascular events. Endothelial cells adjacent to the injured cells proliferate and migrate simultaneously. Peripheral blood circulating EPCs are mobilized to participate in the vascular repair and neovascularization. The flow cytometry results suggest the number EPCs in peripheral blood in hyperhomocysteinemia rats was significantly decreased. Treated with ONOO^-^ scavenger FeTMPyP elevated the number EPCs in peripheral blood. However, the number of EPCs in peripheral blood is low and accounts for only 0.01% cells of the peripheral blood, preventing easy isolation [41,42]. We identified EPCs by counting the circulating CD34^+^/CD31^+^cells. CD34 is a primary marker associated with pro-vasculogenic subpopulation of hematopoietic stem cell (HSCs), while CD31 possesses endothelial characteristics. However, mature endothelial cells also express CD34^+^ and circulating EPCs undoubtedly express a variety of typical endothelium-like markers [43]. Therefore, by equating EPCs with CD34^+^/CD31^+^ cells, our present approach is theoretically defective, partially. Our current data of EPCs numbers were a little higher than theoretical estimates. As a marker of immature cells, CD133 is better than CD34 [44] and only exists in hematopoietic cells. In subsequent experiments, we will select CD133 as a more representative progenitor cells marker. Despite this limitation, further we successfully isolated EPCs from bone marrow of mice, and explored the direct effect of nitrative stress on EPCs by culturing bone marrow-derived EPCs *in vitro*. Our data showed that peroxynitrite decreased the cell survival rate, and induced apoptosis and necrosis of EPCs. The findings in this study establish that peroxynitrite directly damages EPCs. Until now, it is intriguing that the direct effect of nitrative stress on EPCs was not reported. Here, we have shown for the first time that nitrative stress directly contributed to the injury of EPCs.

In a word, this study indicates that nitrative stress play a role in vascular endothelial injury in hyperhomocysteinemia, as well as induce endothelial progenitor cells injury directly. Our data presented here highlight the mechanism of hyperhomocysteinemia-induced endothelial injury, and offer a new insight into the risk factors of cardiovascular disease. Additional experiments will be carried on to explore the specific mechanisms involved in EPCs exposed to nitrative stress.
